# Surface Engineering of the Encapsulin Nanocompartment of *Myxococcus xanthus* for Cell-Targeted Protein Delivery

**DOI:** 10.1021/acsomega.4c10285

**Published:** 2025-02-12

**Authors:** Sac Nicté Gómez-Barrera, Willy Ángel Delgado-Tapia, Aquetzali Estefanía Hernández-Gutiérrez, Maribel Cayetano-Cruz, Carmen Méndez, Ismael Bustos-Jaimes

**Affiliations:** † Departamento de Bioquímica, Facultad de Medicina, Universidad Nacional Autónoma de México, Av. Universidad 3000, CDMX, Mexico 04510, Mexico; ‡ Departamento de Embriología y Genética, Facultad de Medicina, Universidad Nacional Autónoma de México, Av. Universidad 3000, CDMX, Mexico 04510, Mexico

## Abstract

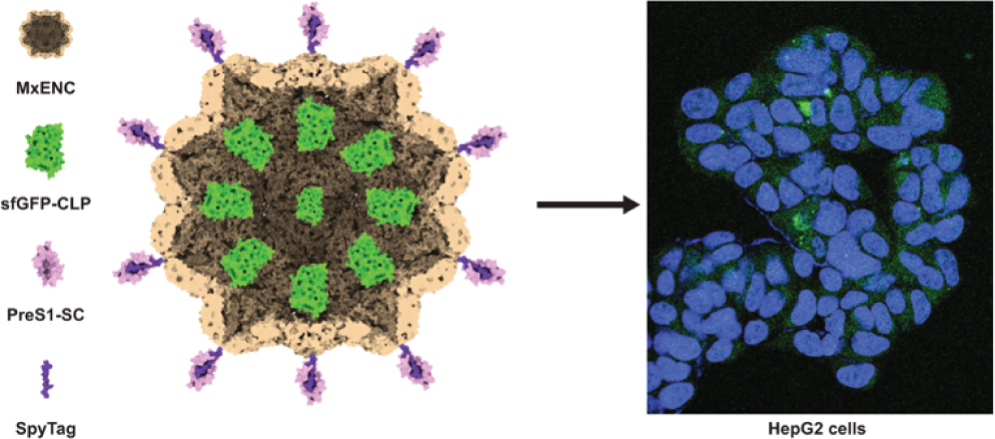

Encapsulin nanocompartments (ENCs), or simply encapsulins, are a novel type of protein nanocage found in bacteria and archaea. The complete encapsulin systems include protein cargoes involved in specific metabolic tasks. Cargoes are selectively encapsulated due to the presence of a specific cargo-loading peptide (CLP). However, heterologous proteins fused to the CLP have also been successfully encapsulated, making encapsulins a very promising system for protein-carrying and delivery. Nevertheless, for precise cell or tissue delivery, encapsulins require the addition of tagging peptides or proteins. In this study, the external surface of the *Myxococcus xanthus* ENC (MxENC) was analyzed and modified to carry the bioorthogonal conjugation peptide (SpyTag) to further decorate the MxENCs with any targeting protein previously fused to the SpyTag orthogonal pair, the SpyCatcher protein. The structural analysis of MxENC led to the selection of the surface loop 155–159 and the C-terminus of the encapsulin shell protein (EncA) for the genetic fusion of the SpyTag peptide. The engineered EncA forms retained the competence for self-assembly into ENCs. To provide cellular specificity, the PreS1_21–47_ hepatocyte-targeting peptide, genetically fused to the SpyCatcher protein, was successfully conjugated to both engineered versions of the MxENC. The modified nanocompartments underwent comprehensive characterization for stability, cargo loading, cellular uptake, and cargo release in HepG2 cells, demonstrating their potential as protein-delivery vehicles. These results provide valuable insights into the design and customization of nanocompartments, opening up possibilities for improved drug delivery applications in biotechnology and nanomedicine.

## Introduction

Continuous progress in drug delivery systems has significantly advanced nanomedicine and biomedical science. Nanoparticles like liposomes, polymers, micelles, and protein cages have been engineered for this purpose.^1,2^ Nanoparticles have proven to be excellent biocompatible carriers, well-received by the body, eliciting appropriate responses and demonstrating little to no toxicity.^3^

Encapsulins are naturally occurring proteins that self-assemble into nanocages termed Encapsulin Nano-Compartments (ENCs). These protein cages are found in diverse prokaryotic microorganisms, i.e., bacteria and archaea.^3,4^ Thanks to an extension sequence in the C-terminus, known as the targeting peptide (TP) or the cargo-loading peptide (CLP), they can encapsulate homologous and heterologous cargoes, making them ideal candidates for constructing nanoscale delivery vehicles for protein-based drugs.^5−7^ In this context, the surface engineering of encapsulins holds excellent potential for cell tagging, thereby enabling the development of tissue-specific delivery vehicles with tailored functionalities,^3,8,9^

This research is focused on the surface modification of the *M. xanthus* ENC (MxENC)^10,11^ as an approach to create targeted delivery systems. The wild-type MxENC comprises the proteins EncA, EncB, EncC and EncD. EncA is the shell protein, while EncB, EncC, and EncD are cargo proteins. EncB and EncC have ferroxidase centers that allow the oxidation of Fe^2+^ to Fe^3+^ for iron storage, whereas Enc D is a NAD(P)H-dependent ferric reductase. The encapsulin system of *M. xanthus* is, therefore, an iron homeostasis system in this bacterium.^12^ The MxENC self-assembles into a *T* = 3 icosahedral symmetry nanocage, constituted of 180 protomers, forming 12 pentameric and 20 hexameric units, resulting in nanocompartments of ∼32 nm diameter.^10^ The self-assembly of EncA is regulated by the folding dynamics of individual polypeptide chains and dynamic noncovalent interactions among these chains, occurring within subunits and at the interfaces between subunits in the resulting supramolecular architecture.^13^

Modifying the surface of ENCs could significantly disturb their cargo-loading capacity and specificity, as well as their cargo-release capabilities, potentially affecting the stability of the nanocompartment.^14−16^ However, this advancement would allow the development of site-specific delivery vehicles with custom-made properties.^17,18^

The surface of ENCs can be modified through chemical or genetic methods to incorporate targeting peptides or proteins. A notable strategy for this purpose is the’plug and display’ approach using conjugating proteins, with a particular focus on the SpyTag-SpyCatcher technology,^19,20^ which is based on the CnaB2 domain from the fibronectin-binding protein FbaB from *Streptococcus pyogenes* (Spy). SpyTag, a 13-residue peptide, naturally forms an isopeptide bond with SpyCatcher, a 115-amino-acid protein, resulting in stable covalently linked complexes. This system has been successfully applied for the decoration of protein nanocages as well as for the fusion of peptides and proteins to gain novel functions.^19,21,22^ Our research uses the SpyTag-SpyCatcher conjugation system to attach the targeting peptide PreS1_21–47_, derived from the Hepatitis B Virus, which has been shown to provide affinity and internalization into hepatocytes,^21,23,24^ This modification at a specific site on the protein monomer allows the introduction of functional groups on the nanocage surface.^8,25^ Careful design of the heterologous insertion and the location in the protein monomer must be performed without disrupting the structure of the cage.^26^ Therefore, the main goal of this study was to produce modified nanocompartments of *M. xanthus* EncA protein, evaluate their stability, and demonstrate their conjugation with the PreS1_21–47_ peptide and internalization into hepatocytes.

In the subsequent sections of this paper, we will delve into the methods employed for encapsulin modification, cargo loading, and characterization. The results obtained from our experiments could lead to a deeper understanding of encapsulin-mediated drug delivery and its potential applications in precision biotechnology and nanomedicine.

## Experimental Section

### Bioinformatic Analysis of the MxENC

The STRIDE server was used to assign secondary structures automatically.^27^ PyMOL 2.0 (Schrödinger, LLC.) and ChimeraX 1.8^28^ provided a visual aid for the structure analysis of MxENC (PDB 4PT2). Surface analysis of the MxENC was carried out with the Virus Particle Explorer database (VIPERdb).^29^

### Chemicals

Ampicillin, Kanamycin, and Isopropyl-β-d-thiogalactoside (IPTG) were purchased from GoldBio, USA. Guanidinium chloride (GuHCl), l-Arg, NaCl, NaH_2_PO_4_, Tris(hydroxymethyl)aminomethane (Tris), MgCl_2_, NH_4_Cl, uranyl acetate, and imidazole were of analytical grade and purchased from Sigma-Aldrich, Mexico. Dulbecco’s Modified Eagle Medium (DMEM) was purchased from Gibco BRL, USA. VectaShield Antifade Mounting Medium was purchased from Vector Laboratories, USA, and 4′,6-diamidino-2-phenylindole (DAPI) was purchased from Thermo Scientific, USA. All other chemicals were of analytical grade.

### Genetic Constructions

All genes were codon-optimized for expression in *E. coli* and chemically synthesized (Gene Universal Inc.). Peptide PreS1_21–47_ coding sequence was fused to the SpyCatcher gene (PreS1-SC). Each gene EncA-157ST and PreS1-SC was directionally cloned in the pET22b(+) vector between restriction sites NdeI and XhoI, generating the plasmids pET22b(+)-EncA-157ST and pET22b(+)-PreS1-SC. EncA-ST gene was directionally cloned in the pET22b(+) vector between restriction sites NdeI and *Eco*RI, generating the plasmid pET22b(+)-EncA-ST. The three constructions include 6xHis tags fused to the C-terminus of each protein. The gene of the heterologous cargo protein, sfGFP-CLP, was directionally cloned in the pET28a(+) vector between restriction sites NdeI and *Eco*RI, generating the construction pET28a(+)-sfGFP-CLP with a N-terminal 6xHisTag. The gene sequences are provided in Table S1.

### Recombinant Protein Production

*E. coli* BL21(DE3) cells were used for recombinant protein expression. Cells were transformed by the heat shock method with the single plasmids (pET22b(+)-EncA-ST, pET22b(+)-EncA-157ST, pET22b(+)-PreS1-SC or pET28a(+)-sfGFP-CLP) or cotransformed with each encapsulin variant (pET22b(+)-EncA-ST or pET22b(+)-EncA-157ST) and the cargo protein (pET28a(+)-sfGFP-CLP). The transformed cells were cultivated in Luria–Bertani (LB) medium supplemented with an appropriate antibiotic (100 μg/mL of ampicillin, 50 μg/mL of kanamycin, or both antibiotics for the coexpression).

Expression experiments were performed in 0.5 L of LB culture medium. The bacteria were grown until the optical density at a λ = 600 nm reached 0.5 at 37 °C. Then, PreS1-SC, sfGFP-CLP, EncA-ST, and the coexpression of EncA-ST with the cargo protein sfGFP-CLP were induced with 0.4 mM IPTG for 6 h at 30 °C. The EncA-157ST and sfGFP-CLP coexpression were induced with 0.4 mM IPTG and cultured for 16 h at 20 °C. Cells were harvested by centrifugation at 8,000 rpm for 20 min at 15 °C and stored at 4 or −70 °C for subsequent analysis.

### Protein Purification

Bacteria pellets were resuspended in an appropriate lysis buffer and disrupted by sonication. Lysis buffer 1 (50 mM HEPES, 20 mM MgCl_2_, 150 mM NH_3_Cl, 200 mM l-Arg, 15 mM Imidazole, pH 7.5) was used for EncA-ST pellet lysis. For EncA-157ST cells, a lysis buffer 2 (50 mM Tris-HCl, 20 mM MgCl_2_, 150 mM NaCl, 200 mM l-Arg, 15 mM Imidazole, pH 7.5, added with PMSF 0.1 mM) was used. Pellets with PreS1-SC and sfGFP-CLP were resuspended in PBS buffer (37 mM NaCl, 2.7 mM KCl, 10 mM Na_2_HPO_4_, and 1.8 mM KH_2_PO_4_) pH 7.5. Cell debris was removed from the lysate by centrifugation at 12,000 rpm for 20 min at 10 °C; all recombinant proteins were recovered in the cleared lysate.

The EncA-modified encapsulins (empty or loaded) were purified by immobilized metal affinity chromatography (IMAC). The supernatants for each protein sample were loaded into a 5 mL HisTrap FF Column (Cytiva) by injection in an Äkta Pure (GE Healthcare) equipment, previously equilibrated with equilibrium buffer (50 mM HEPES pH 7.5, 20 mM MgCl_2_, 150 mM NH_3_Cl, 100 mM l-Arg, and 15 mM Imidazole for EncA-ST encapsulins, or 50 mM Tris-HCl pH 7.5, 20 mM MgCl_2_, 150 mM NaCl, 100 mM l-Arg, and 15 mM Imidazole for EncA-157ST encapsulins). Each HisTrap FF column was washed with 3 volumes of column (CV) of equilibrium buffer, and the bounded protein was eluted by 3 steps (50, 75, 100%) with elution buffer (equilibration buffer with 500 mM imidazole).

For the encapsulins loaded with sfGFP-CLP, size exclusion chromatography (SEC) purification was used to separate the complex of the EncA-ST encapsulin loaded with sfGFP-CLP (EncA-ST:sfGFP) or the complex of the EncA-157ST encapsulin loaded with sfGFP-CLP (EncA-157ST:sfGFP) from the free sfGFP-CLP protein. Thus, the samples obtained from IMAC purification were loaded into a Superdex 200 column in an Äkta Pure equipment pre-equilibrated with PBS buffer at pH 7.5. All protein samples were analyzed using sodium dodecyl sulfate-polyacrylamide gel electrophoresis (SDS-PAGE).

### Multiangle Light Scattering and Dynamic Light Scattering

The hydrodynamic behavior and concentration distribution of the nanocompartments were characterized by dynamic light scattering (DLS) or multiangle light scattering (MALS) with Zetasizer Ultra equipment (Malvern Panalytical). Samples collected from IMAC and SEC purification were filtered through 0.22 μm membrane filters and placed in adequate DLS cuvettes for measurement.

### Zeta Potential Measurement

The Zeta potential of the protein samples was determined using the Zetasizer Ultra (Malvern Panalytical). For measurements at pH 7.4, the protein samples were effectively suspended in a 0.1× PBS buffer, which includes 13.5 mM NaCl, 0.27 mM KCl, 1 mM Na_2_HPO_4_, and 0.18 mM KH_2_PO_4_, demonstrating a conductivity of 4.7 mS/cm. To evaluate the surface charge of both empty and cargo-loaded nanocompartments associated with the histidine tag within the endosomal environment, Zeta potential measurements were conducted at pH 5.5 in 50 mM citrate buffer.

### Native Agarose Gel Electrophoresis (NAGE)

A 0.8% agarose gel was prepared by dissolving 0.16 g of agarose in 20 mL of 1× TAE buffer. The agarose was melted in a microwave oven using 30-s pulses until a homogeneous solution was obtained. The solution was then poured into a gel casting tray equipped with combs to form wells and allowed to cool at room temperature until gel formation. Protein samples were prepared by mixing 5 μL of each sample with 15 μL of 4× loading buffer (95% formamide, 0.05% SDS, 2 mM EDTA, bromophenol blue, and xylene cyanol), resulting in a final volume of 20 μL per sample, which was loaded into the wells. Electrophoresis was carried out at 60 V for 90 min. Afterward, the gel was removed and stained with Coomassie blue for 1 h to visualize the proteins, followed by destaining with water, which was changed several times over 3 days.

### Transmission Electron Microscopy

The morphological characterization of the nanocompartments was performed by TEM with a JEOL-JEM 1200 microscope. The eluted SEC samples were diluted to a concentration of 0.01–0.03 mg/mL, filtered through a 0.22 μm membrane filter, and fixed for 6 min in a copper coated with carbon-Formvar grid. The excess sample was removed by blotting with filter paper. Immediately, the grid was negatively stained with 5 μL Uranyl Acetate 0.5% (pH 5) for 2 min, and the excess was removed by blotting with filter paper.

### Stability of Unloaded Nanocompartments

To assess the stability of chimeric ENCs, the denaturing agent GuHCl and thermally induced disassembly methods were used. Unloaded EncA-ST and EncA-157ST ENCs samples were incubated with GuHCl at final concentrations between 1 and 5 M for 2 h at room temperature with orbital agitation set at 100 rpm. Intrinsic tryptophan fluorescence (ITF) was used to monitor changes in the fluorescence emission spectra and assess the influence of the denaturing agent on the structure of the chimeric ENCs. Furthermore, dynamic light scattering (DLS) was used to monitor any changes in the size of the encapsulin variants. ITF spectroscopy measurements of ENCs at various assembly stages were conducted using a PC1 ISS Spectrofluorometer (Champaign, IL, USA). Emission spectra (λ_exc_ = 280 nm) were acquired at a protein concentration of 0.5 mg/mL, with emission recorded in the 300 to 450 nm range using excitation and emission slit widths of 2 mm. The spectra were subsequently smoothed utilizing the instrument’s software. For thermal stability assays, purified ENCs were incubated for 1 h at 20, 30, 40, or 50 °C in their equilibrium buffers and then analyzed by DLS at their corresponding temperature.

### ENCs Decoration with PreS1-SC

The SpyTag/SpyCatcher bioorthogonal conjugation system was used to attach the PreS1_21–47_ cell-targeting peptide to the surface of the chimeric MxENCs. To achieve this, the PreS1-SC chimera and each chimeric MxENC, which had been previously purified by IMAC, were mixed in a 1:4 molar ratio (monomer of PreS1-SC to EncA-ST or EncA-157ST) and incubated overnight in a Thermomixer (Eppendorf) at 25 °C at 400 rpm. After the incubation, the functionalized particles were purified by SEC, as described before, and SDS-PAGE analysis was performed.

### Internalization Assays of ENCs

The cellular uptake capability of the chimeric ENCs loaded with sfGFP-CLP and decorated with PreS1-SC was assessed using confocal Microscopy. 50,000 HepG2 cells were maintained in 2 mL of Dulbecco’s Modified Eagle Medium (DMEM) in a 35 mm tissue culture dish and grown for 24 h on coverslip at 37 °C. After 24 h, 100 μL of 5 × 10^10^ particles/mL of the decorated and loaded encapsulins were added to the growth medium. Two treatments were added to the cells. Treatment 1 consisted of EncA-ST:sfGFP conjugated to PreS1-SC (EncA-ST:sfGFP + PreS1) and treatment 2 consisted of EncA-157ST:sfGFP conjugated to PreS1-SC (EncA-157ST:sfGFP + PreS1). Then, cells were incubated at 37 °C for 1 h to facilitate binding. The cells were washed 1× with PBS, and 1 mL of DMEM was added to the coverslips. The cells were subsequently incubated at 37 °C for 3, 6, 12, and 24 h. Afterward, the cells were washed 2× with PBS-Trypsin 0.05% and 2× with PBS. Cells were fixed with ice-cold 4% paraformaldehyde in PBS (PFA/PBS) for 15 min at room temperature. After fixing, cells were washed 3× with PBS. The nuclei of cells were stained with 4′,6-diamidino-2-phenylindole (DAPI). Finally, the cells were washed 1× with PBS, mounted in VectaShield, and stored at 4 °C until analyzed by confocal microscopy.

### Fluorescence Change Analysis of Confocal Microscopy Images

ImageJ/Fiji software^30,31^ was used to quantify fluorescence intensity in the images obtained from confocal microscopy over time. To extract fluorescence intensity, a Region of Interest (ROI) was defined in each cell in the frames, and this ROI was applied to all frame sequences obtained for each time point studied. The final value for each point corresponded to the average of the values obtained. A second ROI was selected in an area free of specific fluorescence in each image to minimize background fluorescence. The average intensity of this background ROI was measured in each frame and subtracted from the intensity of the primary ROI, thus obtaining corrected fluorescence values for each frame.^32−34^ Finally, the obtained values were normalized based on the maximum value corresponding to the time of peak fluorescence.

### Proteolysis Assay

In vitro proteolysis experiments were conducted in PBS at pH 7.4 with trypsin. For each assay, 80 μL of encapsulins loaded with sfGFP-CLP corresponding to 7.2 × 10^12^ or 5.1 × 10^12^ particles/mL of EncA-ST:sfGFP or EncA-157ST:sfGFP, respectively, were transferred into Eppendorf tubes. Subsequently, 25 μL of a 0.2% (w/v) trypsin stock solution was added to each sample, and the reaction mixtures were incubated at 20 °C for 0, 10, or 20 min. Proteolytic activity was quenched by rapidly cooling the samples on watered ice for 1 min. Then, the samples were mixed with gel loading buffer and incubated at 92 °C for 15 min before SDS-PAGE analysis.

## Results and Discussion

**Figure 1:**
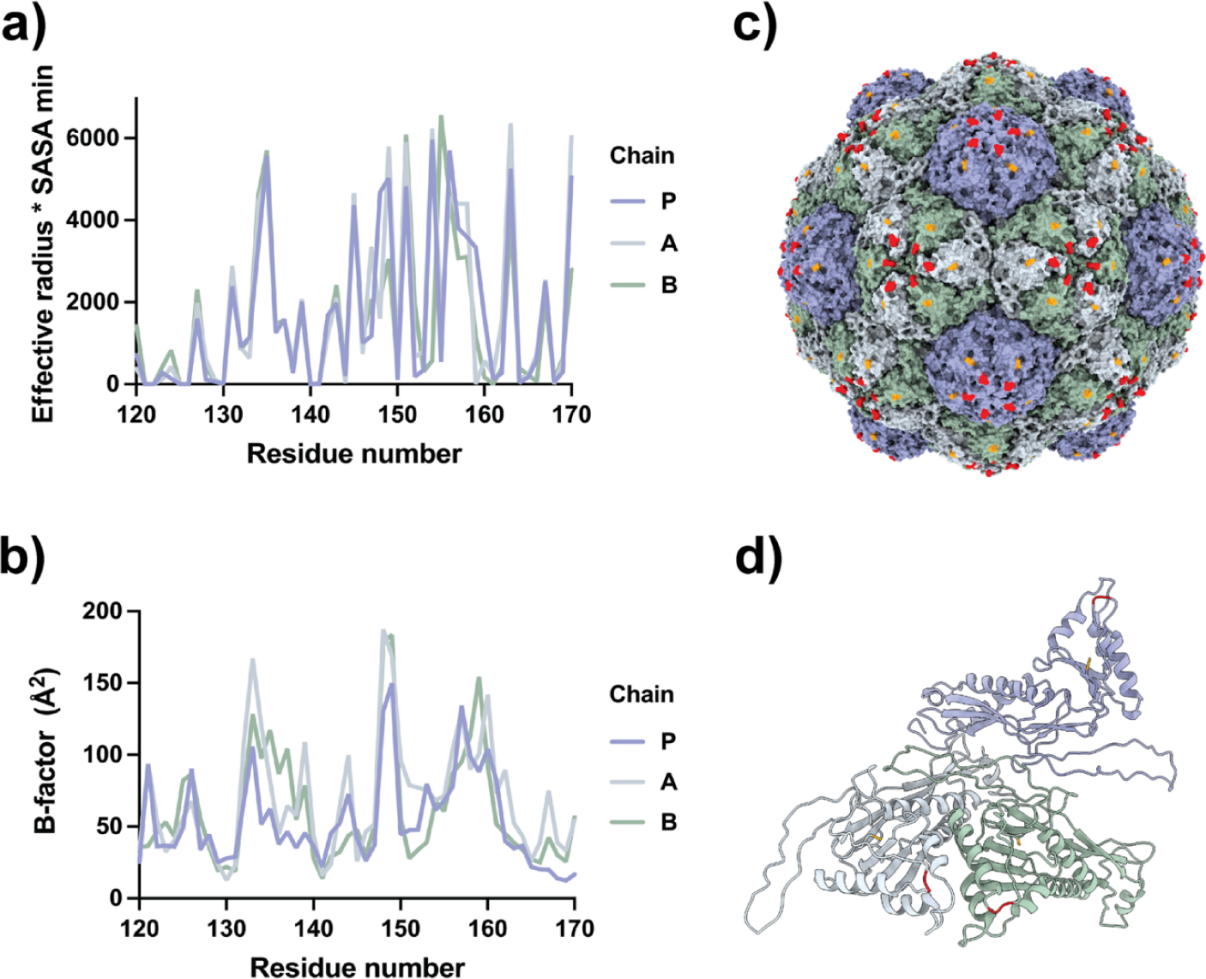
(a) Accessible surface profile for the segment 120–170 of the EncA protein in the MxENC calculated as implemented in the VIPERdb server. The quantity plotted is an amplified SASA (min) value by the effective Radius. SASAmin = SASAmax for the exposed residues. The effective radius corresponds to the radius at which a residue is located minus the inner radius of the particle. (b) B-factors of the segment 120–170 of the EncA protein in the MxENC (PDB 4PT2). (c) The model of the entire MxENC cage shows residues 157 and 158 in red at the apical tip of the loop 131–162. The orange spot corresponds to the last modeled residue of the C-terminal region of the monomeric EncA, Leu276. (d) Cartoon representation of the asymmetric unit of the MxENC, keeping the same colors as in panel (c).

Selection of Superficial Regions of the MxENC for SpyTag Fusion. The structural analysis of MxENC was based on the MxENC Cryo-EM structure described by McHugh and coworkers (PDB 4PT2).^10^ The automatic secondary structure assignment with the STRIDE server revealed that the three EncA chains in the structure are similar but not identical (Figure S1). Visual examination of the particle also revealed the presence of coil-structured tracts disposed on the surface of the particle. One such region comprises residues 131–162, which appear in the three subunits of the asymmetric unit of the structure. Structural data from the VIPERdb server indicated no contacts in residues 137–161. According to the solvent-accessible surface area (SASA) plot analysis provided by VIPERdb, this coil tract is not fully exposed to the solvent (Figure 1a). Regions around residues 133–136 and 155–160 are the most extended solvent-exposed tracts of residues. These residues also show high atomic displacement factors (Figure 1b), suggesting regions with high mobility and, therefore, not critically committed to the structure stability. Considering the whole structure of the MxENC, the most apical region of the coil tract was selected for inserting the SpyTag peptide between residues 157 and 158 (Figure 1c,d). In the designed protein, the inserted peptide was flanked at each side by three Gly residues to promote high mobility; this construction was named EncA-157ST. Michel-Souzy and coworkers introduced a heterologous peptide in loops 138 in *Thermotoga maritima* and 135 in *Brevibacterium linens* encapsulins.^8^ These are protein cages composed of 60 monomers assembled in a *T* = 1 symmetry, while the MxENC has a *T* = 3 symmetry.^10,35^ These residues, 138 and 135 in *T. maritima* and *B. linens* encapsulins, are structural homologues of residue 145 in the *M. xanthus* EncA protein. This position has also been used to insert a 20-mer immunogenic peptide of the SARS-CoV-2 virus spike protein receptor binding domain. However, this specific peptide in this position produced no assembled particles, highlighting the importance of the insertion point as well as the size and composition of the heterologous insert.^36^ This residue is only 12 residues away from the insertion site selected in our research and is part of the extended loop 131–162 spotted here. Another region of interest is the C-termini of EncA; this region is missing in the model, revealing very high mobility. However, the last resolved residue, Leu278, is on the external surface of the ENC, implying that the rest of the chain is also displayed on the external surface of the nanoparticle (Figure 1c,d). Thus, the SpyTag peptide was also fused to the C-terminal of EncA flanked by three and two Gly residues at its *N*- and C-terminus, respectively. This insertion is followed by a TEV protease cutting site and a 6xHisTag to facilitate protein purification; this chimeric protein was named EncA-ST.

### Protein Expression and Purification of Empty and Loaded MxENCs

**Figure 2:**
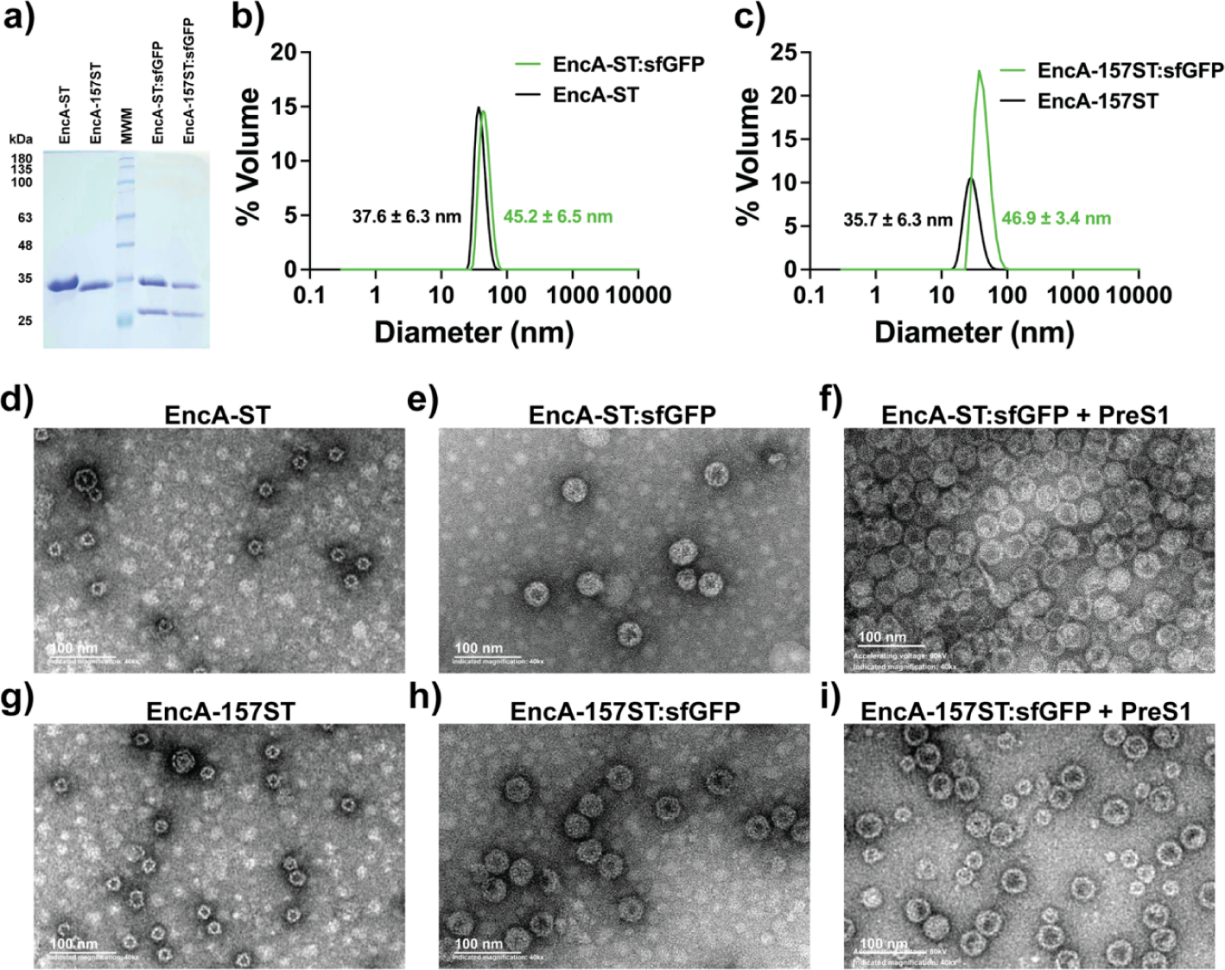
Characterization of the empty, sfGFP-CLP-loaded, and PreS1-decorated ENCs. (a) SDS-PAGE analysis of the IMAC purified EncA-ST and EncA-157ST chimeras (lanes 1 and 2 from left to right), and the IMAC and SEC purified EncA-ST and EncA-157ST chimeras coexpressed with sfGFP-CLP (lanes 4 and 5 from left to right). The fractions shown for the loaded ENCs correspond to the void volume of the SEC column. (b) MALS analysis of the IMAC and SEC purified ENCs of EncA-ST and EncA-ST coexpressed with sfGFP-CLP. The polydispersity indexes (PDI) were 0.113 and 0.024 for EncA-ST and EncA-ST:sfGFP, respectively. (c) MALS analysis of the IMAC and SEC purified ENCs of EncA-157ST and EncA-157ST coexpressed with sfGFP-CLP. PDI were 0.083 and 0.079 for EncA-157ST and EncA-157ST:sfGFP, respectively. (d) TEM analysis of the purified ENCs of EncA-ST. (e) TEM analysis of the purified ENCs of EncA-ST loaded with sfGFP-CLP (EncA-ST:sfGFP). (f) TEM analysis of the purified ENCs of EncA-ST loaded with sfGFP-CLP and conjugated with PreS1-SC (EncA-ST:sfGFP + PreS1). (g) TEM analysis of the purified ENCs of EncA-157ST. (h) TEM analysis of the purified ENCs of EncA-157ST loaded with sfGFP-CLP (EncA-157ST:sfGFP). (i) TEM analysis of the purified ENCs of EncA-157ST loaded with sfGFP-CLP and conjugated with PreS1-SC (EncA-157ST:sfGFP + PreS1).

*E. coli* BL21 (DE3) was used to express EncA-157ST, EncA-ST, sfGFP-CLP and PreS1-SC proteins. The presence of the interest proteins in the soluble fraction of the lysed bacteria was confirmed by SDS-PAGE, showing molecular masses of 36 and 34 kDa for EncA-ST and EncA-157ST, respectively (Figure 2a). However, the analyses of the pellet and soluble fractions revealed that most of the protein remained in the insoluble fraction (results not shown). Therefore, different lysis buffers were tested to improve the solubility of the proteins of interest. We found that each encapsulin variant is more efficiently recovered with a specific buffer formulation, lysis buffer 1 for EncA-157ST, and lysis buffer 2 for EncA-ST. Both lysis buffers are supplemented with 0.2 M l-Arg, which is well-known for its colloidal stabilizing function of proteins. Then, the proteins were purified by IMAC in HisTrap FF (Cytiva) columns. The proteins were eluted with a 375 mM imidazole solution (75% elution buffer). Protein yields are shown in Table 1. Considering that it was demonstrated later that these proteins are assembled into encapsulins, its purification by IMAC demonstrates that the 6xHisTag is exposed on the surface of the self-assembled particles and implies that also the SpyTag peptide was exposed on the external surface of the EncA-ST particles (Figure 2a).

**Table 1:** Recombinant Protein Yields

Protein	Culture volume (L)	Total volume of purified protein obtained (mL)	Protein concentration(mg/mL)	Total protein (mg)
EncA-ST	0.5	15	1.42 ± 0.1	21.3 ± 0.7
EncA-157ST	0.5	15	1.15 ± 0.2	17.2 ± 1.2
EncA-ST:sfGFP	1.0	15	2.97 ± 0.2	44.5 ± 1.8
EncA-157ST:sfGFP	1.0	15	2.18 ± 0.2	32.7 ± 1.4
PreS1-SC	0.5	15	3.24 ± 0.1	48.6 ± 0.4

To produce the loaded ENCs, the protein sfGFP-CLP was coexpressed with each EncA chimera, and the cells were lysed with the same buffers as their empty counterparts. After IMAC purification, the samples were subjected to SEC purification to remove nonencapsulated sfGFP-CLP. The samples’ SDS-PAGE analysis revealed that the protein in the peak corresponding to the ENCs is loaded with the 30 kDa sfGFP-CLP (Figure 2a). SEC fractions corresponding to encapsulin shells showed the typical fluorescence of the encapsulated sfGFP-CLP. However, the samples were analyzed by NAGE to confirm that sfGFP-CLP is captured by the shells. NAGE analysis revealed that free sfGFP-CLP mobility is higher than that of its complexes with EncA-ST or EncA-157ST, confirming its encapsulation. This analysis also shows that encapsulation of this cargo protein does not notoriously modify the electrophoretic mobility of the assembled encapsulins (Figure S2).

DLS analysis of the encapsulins revealed the presence of a predominant particle diameter of 37.6 nm when the encapsulin is empty and a larger diameter of 45.2 nm when it is loaded with sfGFP-CLP (Figure 2b). The same effect was observed for the EncA-157ST variant of the encapsulin, showing diameters of 35.7 and 46.9 nm for the unloaded and loaded particles, respectively (Figure 2c). The size and nature of the species detected by DLS were also analyzed by TEM, confirming that the presence of cargo notably increased the size of these particles (Figure 2d,e,g,h). In contrast to DLS measurements, TEM images revealed that most of the empty encapsulin shells have diameters of ∼24 nm (Figure 2d,g), usually associated with a triangulation number (T number) of *T* = 1 (60 subunits), while the sfGFP-CLP loaded encapsulins have diameters of ∼39 nm, with a *T* = 3 (180 subunits) (Figure 2e,h). This switch in T number has been previously reported for the *M. xanthus* encapsulin shell;^6,10,11^ however, it usually appears in a minor fraction of the encapsulin shells in the wild type encapsulin. Here, the modifications in EncA disrupted most of the assembly of native *T* = 3 shells, as previously observed for other engineered variant of this encapsulin.^6^ Here, this observed size homogeneity in both protein variants suggests that it might arise from a common trait: the elongated C-terminus. In EncA-ST, the SpyTag peptide is fused to the C-terminus, while in EncA-157ST there is a 6xHisTag fused with the C-terminus of the protein. While this C-terminus elongation could be the trigger for T number switching, it is clear that cargo loading reverted this phenomenon.

### Stability of MxENCs

Although the loop 155–160 and the C-terminal region of EncA in the assembled nanocompartments appeared not to be a compromising region for integrating the SpyTag peptide without disturbing the structure of the ENCs, experimental evidence was collected by subjecting the unloaded particles to chemical and thermal denaturing conditions. For this purpose, the unloaded nanocompartments were exposed to GuHCl (0–5 M) as a chaotropic agent, and their structural modifications were monitored using Intrinsic Tryptophan Fluorescence (ITF) and Dynamic Light Scattering (DLS). In a previous study, Boyton and coworkers conducted the disassembly/assembly characterization of unloaded and unmodified MxENCs by ITF, producing insightful results.^13^ Our research also used ITF as an intrinsic probe of structural changes associated with disassembly and possibly unfolding, disturbing the microenvironments of Trp residues and modifying their emission spectra. When Trp residues are buried within the protein structure and not solvated, the maximum emission wavelength peaks appear at lower values in the emission spectra. If a structural change produces the exposition of such Trp residues to the aqueous solvent, the wavelength of maximum emission shifts to larger wavelengths values, a phenomenon known as red-shift or bathochromic shift.^37^

**Figure 3:**
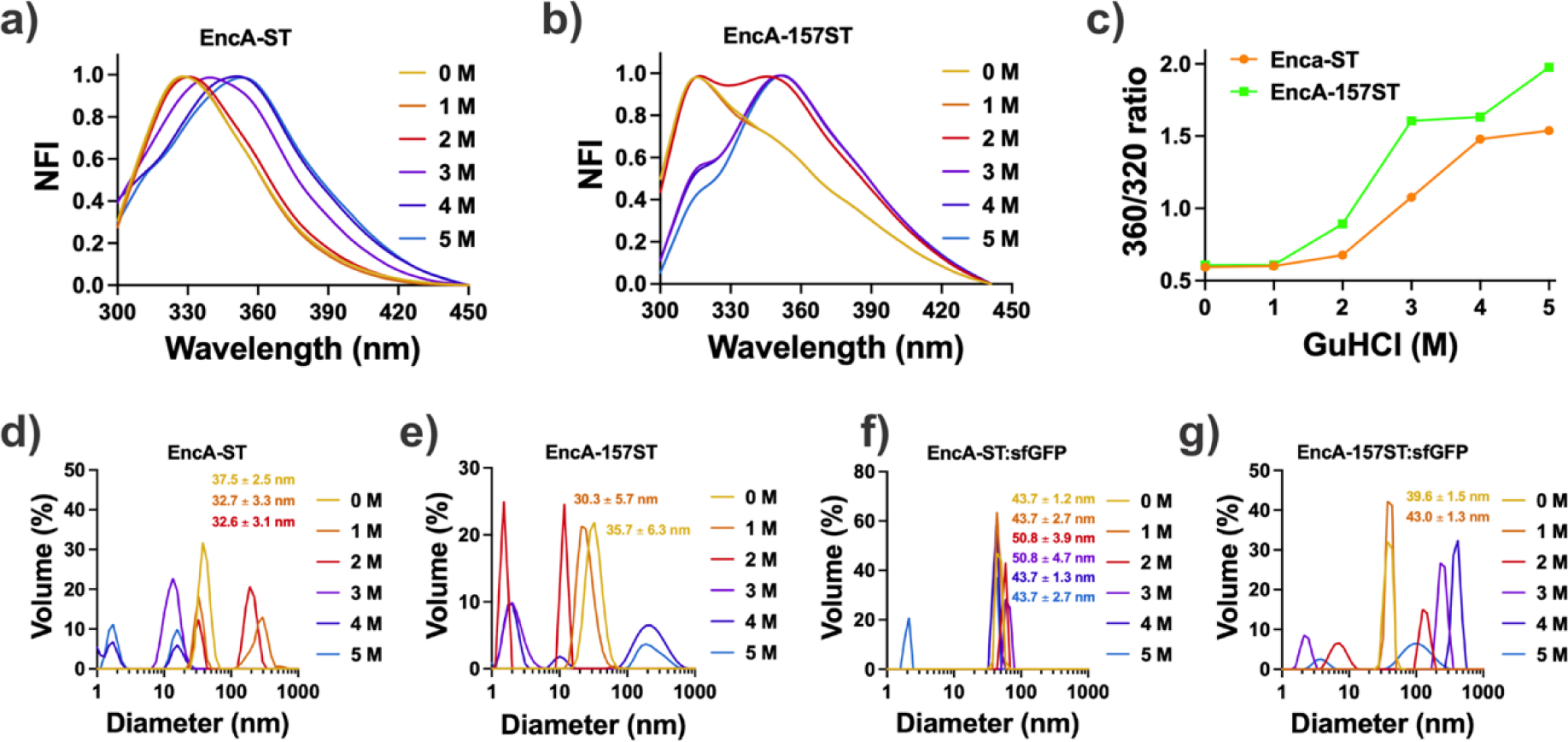
GuHCl-induced disassembly of ENCs. Normalized fluorescence intensity (NFI) emission spectra of (a) EncA-ST ENCs and (b) EncA-157ST ENCs with increasing concentrations of GuHCl. (c) Ratio of the fluorescence intensities at wavelengths of 360 and 320 nm of EncA-ST (orange) and EncA-157ST (green). DLS analysis of encapsulins with increasing concentrations of GuHCl: (d) EncA-ST ENCs (PDI were 0.156, 0.294, 0.245, 0.517, 0.543, and 0.672 for GuHCl concentrations of 0, 1, 2, 3, 4, and 5 M, respectively), (e) EncA-157ST ENCs (PDI were 0.172, 0.319, 0.347, 0.391, 0.612, and 0.757 for GuHCl concentrations of 0, 1, 2, 3, 4, and 5 M, respectively), (f) EncA-ST:sfGFP ENCs (PDI were 0.016, 0.163, 0.499, 0.492, 0.554, and 0.601 for GuHCl concentrations of 0, 1, 2, 3, 4, and 5 M, respectively), and (g) EncA-157ST:sfGFP (PDI were 0.066, 0.069, 0.484, 0.444, 0.561, and 0.593 for GuHCl concentrations of 0, 1, 2, 3, 4, and 5 M, respectively). In panels (d–g), diameter sizes are shown only for species in the range of ENCs.

The EncA protein of *M. xanthus* contains three Trp residues, Trp17 and Trp96, located at interfaces between protomers and Trp155 in a hydrophobic region next to one of the insertion places of the SpyTag peptide (between residues 157 and 158). It has been found that the emission spectra of Trp residues in MxENC show a bathochromic shift upon disassembly, providing an excellent probe for this process.^13,38^ As expected, when the nanocompartments of our variants were exposed to the chaotropic agent GuHCl, the emission spectrum of EncA-ST ENCs also presented a bathochromic shift starting at 3 M GuHCl, and the shift is intensified with the concentration of GuHCl. This strongly suggests that GuHCl at concentrations up to 2 M does not affect the structure of the EncA-ST ENCs. However, from 3 M GuHCl onward, the disassembly of the nanocompartments begins to be observed, and the increase in the spectral red shift stops at 5 M GuHCl. The total change in emission maxima shifted from ∼320 to ∼360 nm (Figure 3a). This result agrees with previous research indicating that modifications at the C-terminal end of the EncA protein do not alter the stability of the particle.^13^ In contrast, the EncA-157ST variant spectrum shows a shoulder at ∼360 nm without GuHCl (Figure 3b). This shoulder increases with GuHCl concentration, also producing a bathochromic shift in the spectra, and suggests that the heterologous peptide insertion modifies the electronic environment of Trp155. Considering the peak at 320 as a representative signal of the native state of the protein and the peak at 360 nm as a characteristic signal of the unfolded protein, the change in the 360/320 normalized fluorescence intensity (NIF) ratio can reveal protein unfolding. The observed change of this ratio with GuHCl (Figure 3c) suggests that the variant EncA-157ST is less stable than EncA-ST, as this ratio increases more notoriously in the former than in the latter. However, the appearance of the peak at ∼360 nm does not guarantee the disassembly and unfolding of the ENCs. Hence, the samples were analyzed by DLS to assess the existing number and size of species. In agreement with the ITF spectra analysis, GuHCl concentrations up to 3 M produce species with a diameter close to that of the ENCs for the variant with the SpyTag at the C-terminus (Figure 3d). Meanwhile, the variant EncA-157ST retained sizes close to that of ENCs only at concentrations of GuHCl between 0 and 1 M (Figure 3e). This finding suggests that the stability of the nanocompartment is slightly compromised when this loop of the EncA is modified, likely due to the hydrophobic nature of the residues in the vicinity of the insertion point, which forms a small hydrophobic pocket in the protein structure.

To examine the effect of cargo loading on encapsulins stability, the loaded particles were also incubated with GuHCl, and the size of species was analyzed by DLS. The effect of the cargo on the stability is clearly illustrated in the EncA-ST:sfGFP complex, whose diameter change was minimal under the assay conditions (Figure 3f), contrasting with the stability shown by its unloaded form (Figure 3d). In opposition, the stability of EncA-157ST:sfGFP complex was not improved by the presence of the loaded protein (Figure 3g), as its stability remained similar to the unloaded encapsulin (Figure 3e). It is worth mentioning that this effect on stability may also depend on the nature of the cargo protein. Cargo proteins have their stabilities and may be loaded in different quantities inside the encapsulin variants. Moreover, cargoes may have excellent, neutral, or even negative packing interactions among themselves and with the coat encapsulins, resulting in an increased or decreased stability of the whole complex. A recent study reports structural heterogeneity provoked by heterologous cargo loading in the *M. xanthus* encapsulin shell.^6^ Thus, cargo proteins might impact the overall structure of the encapsulin, changing its T number and imposing steric hindrance to the assembled complex that will influence the energy of interaction among subunits. Those findings underscore the importance of examining the physicochemical stability of the modified and loaded encapsulins for any future application.

### Thermal Stability of Modified Unloaded MxENCs

MALS analysis of ENCs of the two variants produced in this work, stored at room temperature, showed no diameter changes for at least one year (Figure S3). Previous work revealed that the irreversible disassembly temperature of *M. xanthus* ENCs is 60 °C.^13,39^

As part of our focus on evaluating the thermal stability of our variants, especially under biological conditions, we conducted tests at four different temperatures below the temperature of irreversible disassembly: 20, 30, 40, and 50 °C for 1 h and its colloidal stability was assessed by DLS. No significant variation was found in the size of the EncA-ST nanocages according to DLS analysis (Figure S4). However, in EncA-157ST nanoparticles, minimal aggregation was observed at 40 °C, and a notorious increase in diameter occurred at 50 °C. These findings indicate that both variants of the nanocages exhibit good thermal stability below 40 °C and suggest that they can be used for therapies at the human body temperature.

### Decorated MxENCs as Specific Delivery Vectors

**Figure 4:**
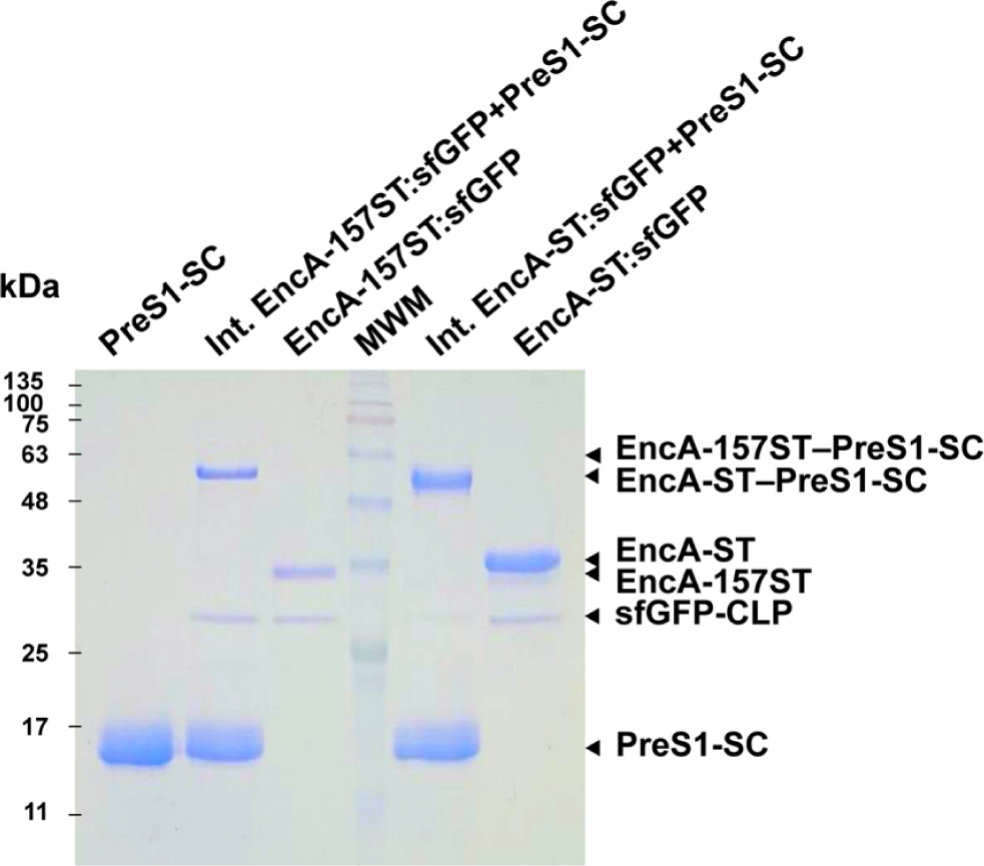
SDS-PAGE analysis of the bioconjugation reaction between PreS1-SC (first lane left to right) and EncA-157ST:sfGFP (third lane), or between PreS1-SC and EncA-ST:sfGFP (sixth lane). Bioconjugation species, EncA-ST–PreS1-SC and EncA-157ST–PreS1-SC, are shown in the second and fifth lanes.

To demonstrate the practical application of the surface-modified MxENCs in specific targeting, the sfGFP-CLP-loaded nanoparticles (Figure 2e,h) were decorated with the peptide PreS1_21–47_ through bioorthogonal conjugation with the SpyTag/SpyCatcher system. The PreS1 peptide was genetically fused to the N-terminus of the SpyCatcher protein, expressed, and purified. Then, it was used for bioconjugation with EncA-ST:sfGFP and EncA-157ST:sfGFP to produce EncA-ST:sfGFP + PreS1 and EncA-157ST:sfGFP + PreS1 nanoparticles. SDS-PAGE analysis of MxENCs, PreS1-SC, and the combination of the bioconjugating partners revealed the presence of the bioconjugation products, EncA-ST–PreS1-SC and EncA-157ST–PreS1-SC (Figure 4). As a confirmative proof of encapsulin decoration with PreS1-SC, the Z-potential of all particles was assessed (Table 2). Z-potential was measured at neutral and acidic pH, considering that the journey from outside the cell to the lysosome would include a drop in pH. It is notable that the change in Z-potential from negative to positive when pH shifts from 7.4 to 5.5. This change in Z-potential may promote the lysosomal escape of the particles, as observed for other particles showing positive Z-potential,^40^ and deserves further study. Finally, to evaluate the effect of the decoration of the modified encapsulin shells on their structure or size, the PreS1-SC conjugated particles were imaged by TEM. This analysis shows that the decoration of EncA-ST:sfGFP with PreS1-SC (Figure 2f) does not visibly alter the structure of the parental particles (Figure 2e). On the other hand, the TEM analysis of the conjugated encapsulins EncA-157ST:sfGFP + PreS1 revealed the presence of two particle populations, one minor population of small particles, presumably *T* = 1, and a major population of large particles (Figure 2i) that remained of the size of the nonconjugated encapsulins (Figure 2h). The small particles may arise from the partial disassembly of the large particles, as the results presented in this research demonstrate a low stability of these encapsulins that, under the conjugation conditions, may have been disassembled to transit from *T* = 3 to *T* = 1 particles, possibly losing their cargoes.

**Table 2:** Zeta Potential of Unloaded and sfGFP-Loaded Encapsulins at Neutral and Acidic pH

Sample	pH	Zeta potential (mV)	Standard deviation (mV)	Conductivity (mS/cm)
EncA-ST	7.4	–4.290	0.259	4.70
EncA-157ST	7.4	–4.725	0.539	3.88
EncAST:sfGFP	7.4	–5.334	0.307	4.13
EncAST:sfGFP + PreS1	7.4	–6.120	0.559	1.73
EncA-157ST:sfGFP	7.4	–4.484	0.414	3.72
EncA-157ST:sfGFP + PreS1	7.4	–5475	0.388	4.95
EncA-ST	5.5	12.560	2.788	22.91
EncA-157ST	5.5	4.908	1.027	15.77
EncAST:sfGFP	5.5	2.403	1.005	17.04
EncAST:sfGFP + PreS1	5.5	10.72	1.889	19.34
EncA-157ST:sfGFP	5.5	1.477	0.373	17.80
EncA-157ST:sfGFP + PreS1	5.5	8.654	2.734	22.91

### Internalization Assays of ENCs

**Figure 5:**
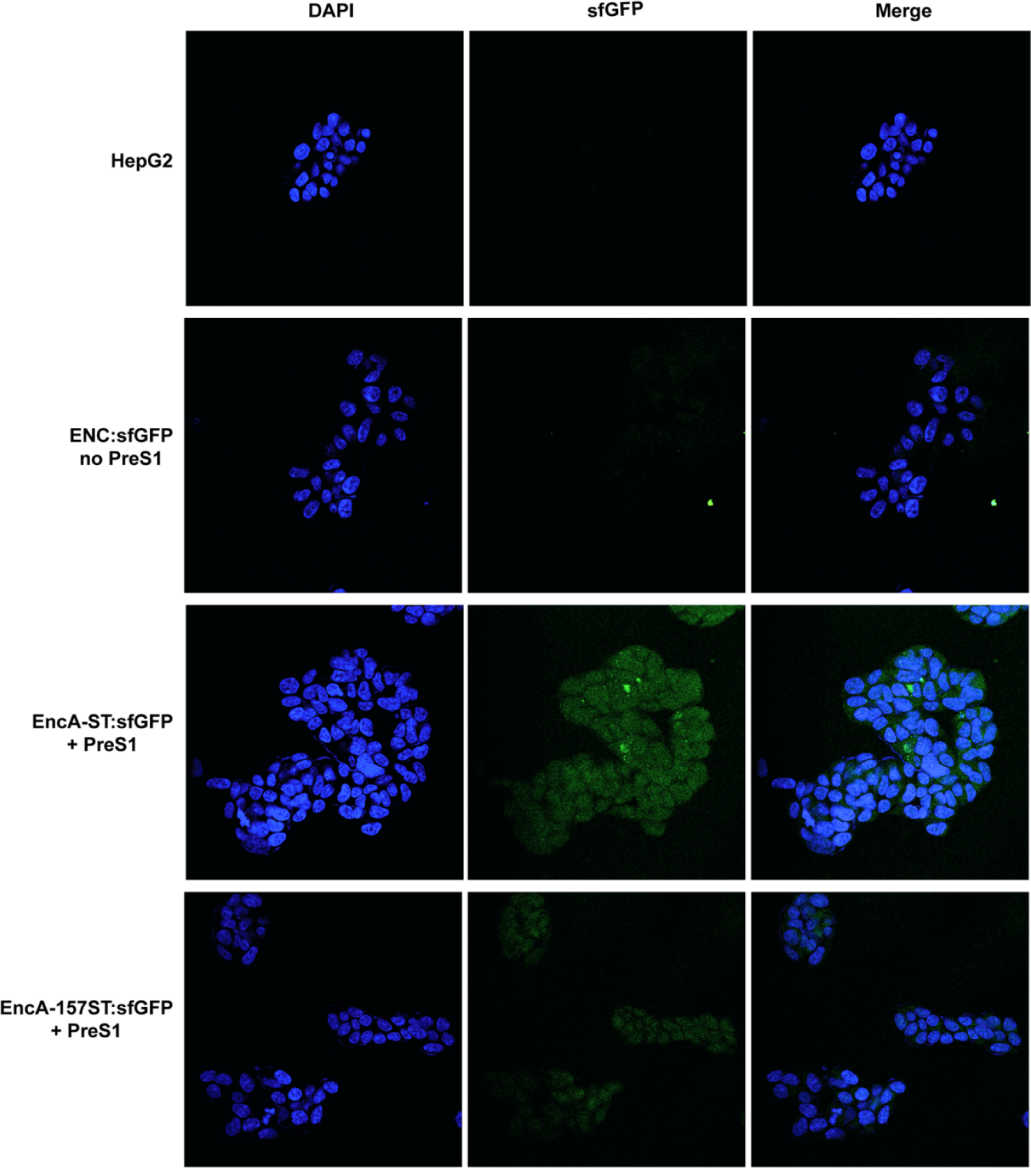
Confocal imaging of HepG2 cells after 3 h of treatment. First row, HepG2 in DMEM. Second row, cells treated with 2.5 × 10^9^ particles/mL of EncA-ST:sfGFP (particles without PreS1 peptide). Third row, cells treated with 2.5 × 10^9^ particles/mL of EncA-ST:sfGFP + PreS1 (treatment 1), and fourth row, cells treated with 2.5 × 10^9^ particles/mL of EncA-157ST:sfGFP + PreS1 (treatment 2). DAPI fluorescence (excitation, 504 nm; emission, >523 nm) and GFP fluorescence (excitation, 405 nm; emission, >488 nm).

Peptide PreS1_21–47_ mediates HBV-specific binding and internalization into hepatocytes.^23,41^ Bchini and coworkers discovered that the HepG2 cell line possesses receptors for HBV attachment and entry.^42^ In recent years, this peptide has been presented on protein nanocages for targeted delivery to hepatic cells.^24,43^ The uptake of ENC variants by HepG2 cells was monitored by confocal fluorescence microscopy using the sfGFP-CLP inside the nanoparticles as a reporter protein for detecting and localizing encapsulin variants in HepG2 cells. Nontreated HepG2 cells and sfGFP-loaded but nondecorated MxENCs were used as negative controls of the experiment. In a previous study, Cayetano-Cruz and coworkers demonstrated that the VLPs decorated with PreS1 are internalized into cells after 3 h of treatment.^44^ In addition to internalization into cells, cargo delivery demands the nanoparticle shell’s disassembly to release the cargo into the cell. The events occurring from the internalization through the early endosome to the lysosome formation include several changes in the environment of the encapsulins, including its exposition to proteases. Therefore, to model the differential proteolytic decay of encapsulins and sfGFP-CLP, changes in the electrophoretic pattern of the encapsulin complexes due to trypsin proteolysis were evaluated. The SDS-PAGE analysis of the treated encapsulins shows that EncA-ST is partially degraded before sfGFP-CLP. At the same time, this effect is not apparent for EncA-157ST within the time explored in this experiment (Figure S5). While trypsin is not an endosomal protease, and endosomal and lysosomal proteases will have different specificities for protein substrates, it is reasonable that cargoes could be released from the encapsulins under endosome-lysosome conditions. Therefore, it is important to determine the approximate time it takes for cargo to be released from the internalized nanocompartments within the cell. Here, we used the fluorescence fading time as a probe for protein release, considering that the protein shell will be proteolyzed by the host cellular proteases releasing the sfGFP-CLP, which will then be proteolyzed and concurrently lose its fluorescence. To address this, we conducted incubation tests at four different time intervals: 3, 6, 12, and 24 h. After the incubation, the cells were washed and fixed, and the nucleic acids were stained with DAPI. Confocal microscopy images of treated cells demonstrated the localization of sfGFP fluorescence inside the cell boundaries (Figure 5).

**Figure 6:**
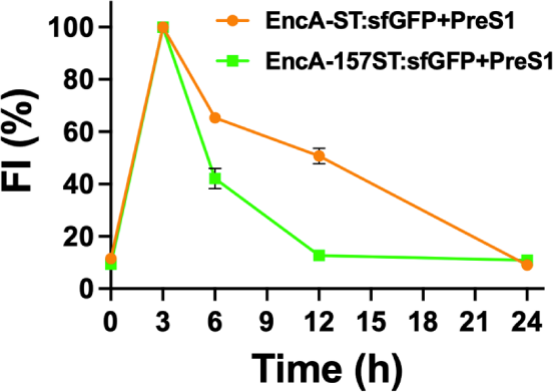
Time course of the normalized fluorescence intensity of the EncA-ST:sfGFP + PreS1 and EncA-157ST:sfGFP + PreS1 internalized into HepG2 cells. The results of three independent experiments and their corresponding SD are plotted.

No fluorescence was observed inside the cells in the nontreated control, and only marginal fluorescence appeared in cells treated with nondecorated ENCs. The total fluorescence was analyzed in the confocal microscopy images at time intervals ranging from 0 to 24 h (Figures S6–S8), revealing an initial increase in fluorescence intensity within the cell boundaries at the first three hours, followed by a sustained decrease of the sfGFP-CLP fluorescence (Figure 6). This observation is fundamental as it may imply the release of the sfGFP-CLP from the encapsulin through the action of proteases, followed by the fading of its fluorescence also produced by proteolytic decay. Notably, the rate of fluorescence intensity loss was remarkably higher for the EncA-157ST variant than for the EncA-ST variant, which is consistent with the lower stability shown by this variant. Moreover, it is worth noting that by 24 h, the fluorescent signal nearly disappears completely for both variants.

## Conclusions

MxENCs are a highly appealing system for protein delivery given its excellent ability to encapsulate CLP-tagged proteins. In this research it was demonstrated that the external surface of MxENCs can be engineered to carry additional motifs for bioconjugation. Moreover, adding orthogonal bioconjugating elements allows the attachment of other proteins that can confer cell-tagging properties. Herein, MxENCs gained access to hepatic cells by including an HBV peptide; similar strategies can be used to internalize ENCs into other tissues, either healthy or ill. Tests for physical-chemical stability revealed that the modified nanocompartments respond differently to denaturing agents and thermodynamic disassembly conditions, emphasizing the importance of studying their stability. The EncA-157ST variant with the PreS1 peptide showed an enhanced cargo release rate, indicating that this rate can also be engineered through protein modifications for potential applications in controlled drug release. Moreover, lysosome escape can also be engineered to warrant that cargo proteins can reach the cytoplasm of target cells to carry out their goals. Our findings provide valuable insights into the structural modification of MxENCs. They present a promising avenue for functionalizing their surfaces, enhancing their potential utility in various biomedical and nanotechnological applications, especially as a drug delivery system.

## Supporting Information Available

Protein sequences of the genes coding for the proteins used in the research; graphical secondary structure assignment for the asymmetric unit of the encapsulin protein (PDB 4PT2); native agarose gel electrophoresis analysis of loaded and unloaded encapsulin shells; long-term stability of the encapsulins at room temperature assessed by MALS; stability of encapsulins at 20, 30, 40, and 50 °C for 1 h assessed by MALS; SDS-PAGE analysis of the time course of the proteolytic degradation of the loaded encapsulin shells; confocal image controls and time-course changes in fluorescence of the sfGFP inside HepG2 cells (PDF)
