# Supplementary material for: Surface Engineering of the Encapsulin Nanocompartment of Myxococcus xanthus for Cell-Targeted Protein Delivery
Source: ACS Omega. 2025 Feb 12;10(7):7142–52. doi: 10.1021/acsomega.4c10285 (PMC11866011; doi:10.1021/acsomega.4c10285)
Supplement: Supplementary file 1 — ao4c10285_si_001.pdf [file ao4c10285_si_001.pdf]

Supporting Information

Surface engineering of the encapsulin nanocompartment of *Myxococcus xanthus* for cell-targeted protein delivery

Sac Nicté Gómez-Barrera[1], Willy Ángel Delgado-Tapia[1], Aquetzali Estefanía Hernández-Gutiérrez[1], Maribel Cayetano-Cruz[1], Carmen Méndez[2], Ismael Bustos-Jaimes[1]*

[1] Departamento de Bioquímica, Facultad de Medicina, Universidad Nacional Autónoma de México, Av. Universidad 3000, CDMX 04510, Mexico.

[2] Departamento de Embriología y Genética, Facultad de Medicina, Universidad Nacional Autónoma de México, Av. Universidad 3000, CDMX 04510, Mexico.

*Corresponding author. E-mail: ismaelb@unam.mx

Table S1. Gene sequences for the chimeric proteins.

**> EncA157-ST**<br>ATGCCGCTGGAACCGCATTTTATGCCGGATTTTCTGGGTCATGCAGAAAATCCGCTGCGCGAAGAAGAATGGGCCC<br>GTCTGAATGAAACCGTTATTCAGGTGGCCCGCCGCAGCCTGGTTGGCAGACGTATTCTGGATATCTATGGCCCGCT<br>GGGTGCCGGCGTGCAGACCGTGCCTTATGATGAATTTCAGGGTGTGAGCCCGGGTGCAGTGGATATTGTTGGTGAA<br>CAGGAAACCGCCATGGTGTTTACCGATGCACGTAAATTCAAAACCATTCCGATTATCTATAAGGATTTCCTGCTGC<br>ATTGGCGTGATATTGAAGCAGCACGCACCCATAATATGCCGCTGGATGTGAGCGCCGCAGCAGGTGCAGCAGCCCT<br>GTGCGCTCAGCAGGAAGATGAACTGATTTTCTATGGCGATGCCCGCCTGGGTTATGAAGGTCTGATGACCGCCAAT<br>GGTCGCCTGACCGTGCCGCTGGGTGACTGGACCAGCGGCGGTGGTGGTGCCCATATTGTTATGGTTGATGCATATA<br>AACCGACCAAAGGCGGCGGCGGTCCGGGTGGTGGTTTTCAGGCCATTGTGGAAGCAACCCGTAAACTGAATGAACA<br>GGGTCATTTTGGTCCGTATGCAGTTGTGCTGAGCCCGCGTCTGTATAGCCAGCTGCATCGCATCTATGAAAAAACC<br>GGCGTTCTGGAAATTGAAACCATTCGCCAGCTGGCCAGCGATGGTGTGTATCAGAGCAATCGTCTGCGCGGTGAAA<br>GCGGCGTTGTGGTGAGCACCGGCCGCGAAAATATGGATCTGGCAGTGAGTATGGATATGGTTGCAGCATATCTGGG<br>CGCCAGTCGCATGAATCATCCGTTTCGCGTTCTGGAAGCACTGCTGCTGCGCATTAAGCATCCGGATGCAATTTGT<br>ACCCTGGAAGGCGCAGGCGCAACCGAACGTCGCCTCGAGCACCACCACCACCACCACTGA
**> EncA-ST**<br>ATGCCGCTGGAACCGCATTTTATGCCGGATTTTCTGGGTCATGCCGAAAATCCGCTGCGTGAAGAAGAATGGGCAC<br>GCCTGAATGAAACCGTTATTCAGGTGGCCCGTCGTAGCCTGGTTGGCCGCCGTATTCTGGATATCTATGGTCCGCT<br>GGGTGCAGGCGTTCAGACCGTTCCGTATGATGAATTTCAGGGTGTGAGCCCGGGCGCAGTTGATATTGTTGGCGAA<br>CAGGAAACCGCAATGGTTTTTACCGATGCCCGTAAATTCAAAACCATTCCGATTATCTATAAGGATTTCCTGCTGC<br>ATTGGCGCGATATTGAAGCCGCCCGTACCCATAATATGCCGCTGGATGTTAGCGCAGCAGCCGGTGCAGCAGCCCT<br>GTGCGCTCAGCAGGAAGATGAACTGATTTTCTATGGCGATGCACGCCTGGGTTATGAAGGCCTGATGACCGCAAAT<br>GGCCGCCTGACCGTTCCGCTGGGTGACTGGACCAGTCCGGGCGGTGGCTTTCAGGCCATTGTGGAAGCAACCCGCA<br>AACTGAATGAACAGGGCCATTTTGGTCCGTATGCAGTTGTGCTGAGCCCGCGTCTGTATAGTCAGCTGCATCGCAT<br>CTATGAAAAAACCGGTGTGCTGGAAATTGAAACCATTCGCCAGCTGGCAAGCGATGGTGTTTATCAGAGCAATCGT<br>CTGCGTGGTGAAAGCGGCGTTGTTGTTAGTACCGGTCGCGAAAATATGGATCTGGCAGTGAGCATGGATATGGTTG<br>CCGCCTATCTGGGCGCAAGTCGTATGAATCATCCGTTTCGCGTGCTGGAAGCCCTGCTGCTGCGTATTAAGCATCC<br>GGATGCCATTTGCACCCTGGAAGGCGCAGGTGCCACCGAACGCCGTCTGGAAGGCGGTGGCGGCGCTCATATTGTT<br>ATGGTTGATGCATATAAACCGACCAAAGGTGGTGAAAATCTGTATTTTCAGAGTGGCGGCCATCATCATCATCACC<br>ATTAA
**> PreS1-SC**<br>ATGCCGCTGGGCTTTTTCCCGGATCATCAGCTCGATCCGGCCTTTGGCGCAAATAGTAATAATCCGGATTGGGATT<br>TTAACCCGGGTGGATCCGGTGGTGTGGATACCCTGAGCGGTCTGAGCAGCGAACAGGGCCAGAGCGGTGACATGAC<br>CATTGAAGAAGATAGCGCCACCCATATTAAGTTTAGTAAACGTGATGAAGACGGTAAAGAACTGGCAGGTGCCACC<br>ATGGAACTGCGCGATAGCAGCGGTAAAACCATTAGTACCTGGATTAGCGATGGTCAGGTTAAAGATTTTTATCTGT<br>ATCCGGGTAAATACACCTTTGTGGAAACCGCAGCCCCGGATGGTTATGAAGTGGCCACCGCCATTACCTTTACCGT<br>TAATGAACAGGGCCAAGTTACCGTGAATGGTAAAGCAACCAAAGGCGATGCACATATTCTCGAGCACCACCACCAC<br>CACCACTGA
**> sfGFP-CLP**<br>ATGCGTAAAGGTGAAGAACTGTTCACCGGTGTTGTTCCGATCCTGGTTGAACTGGATGGTGATGTTAACGGCCACA<br>AATTCTCTGTTCGTGGTGAAGGTGAAGGTGATGCAACCAACGGTAAACTGACCCTGAAATTCATCTGCACTACCGG<br>TAAACTGCCGGTTCCTTGGCCGACTCTGGTGACTACCCTGACCTATGGTGTTCAGTGTTTTGCTCGTTACCCGGAT<br>CACATGAAGCAGCATGATTTCTTCAAATCTGCAATGCCGGAAGGTTATGTACAGGAGCGCACCATTTCTTTCAAAG<br>ACGATGGCACCTACAAAACCCGTGCAGAGGTTAAATTTGAAGGTGATACTCTGGTGAACCGTATTGAACTGAAAGG<br>CATTGATTTCAAAGAGGACGGCAACATCCTGGGCCACAAACTGGAATATAACTTCAACTCCCATAACGTTTACATC<br>ACCGCAGACAAACAGAAGAACGGTATCAAAGCTAACTTCAAAATTCGCCATAACGTTGAAGACGGTAGCGTACAGC<br>TGGCGGACCACTACCAGCAGAACACTCCGATCGGTGATGGTCCGGTTCTGCTGCCGGATAACCACTACCTGTCCAC<br>CCAGTCTGTTCTGTCCAAAGACCCGAACGAAAAGCGCGACCACATGGTGCTGCTGGAGTTCGTTACTGCAGCAGGT<br>ATCACGCACGGCATGGATGAGCTCTACAAAGGTGGTTCTGGTGGATCCGGCGGTAGCGGTGGTCTGACCGTGGGCA<br>GCCTGCGCCGCTGA

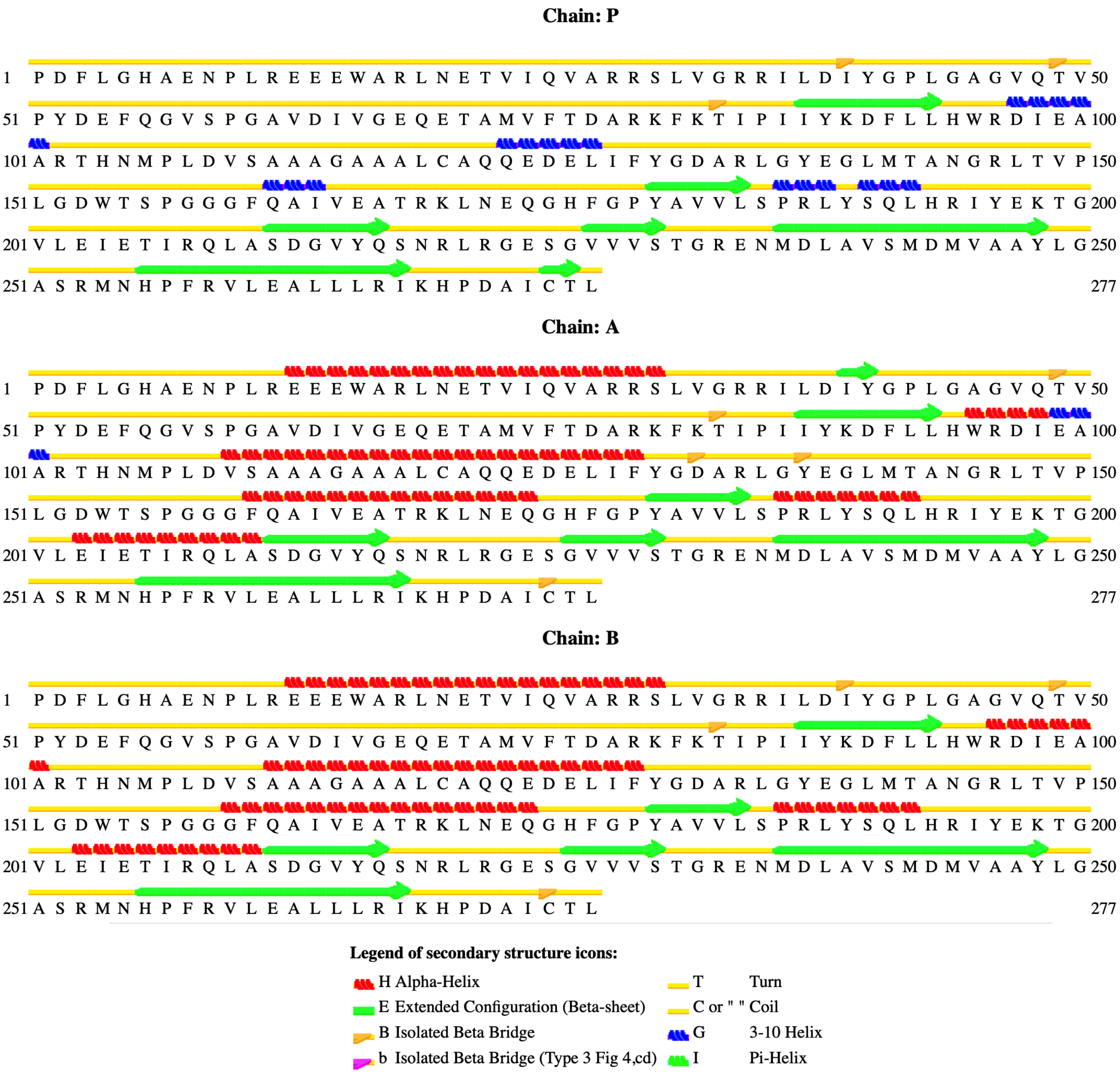

Figure S1. Automatic secondary structure assignment by the STRIDE server (https://webclu.bio.wzw.tum.de/cgi-bin/stride/stridecgi.py) for the asymmetric unit of the MxENC (PDB 4PT2).

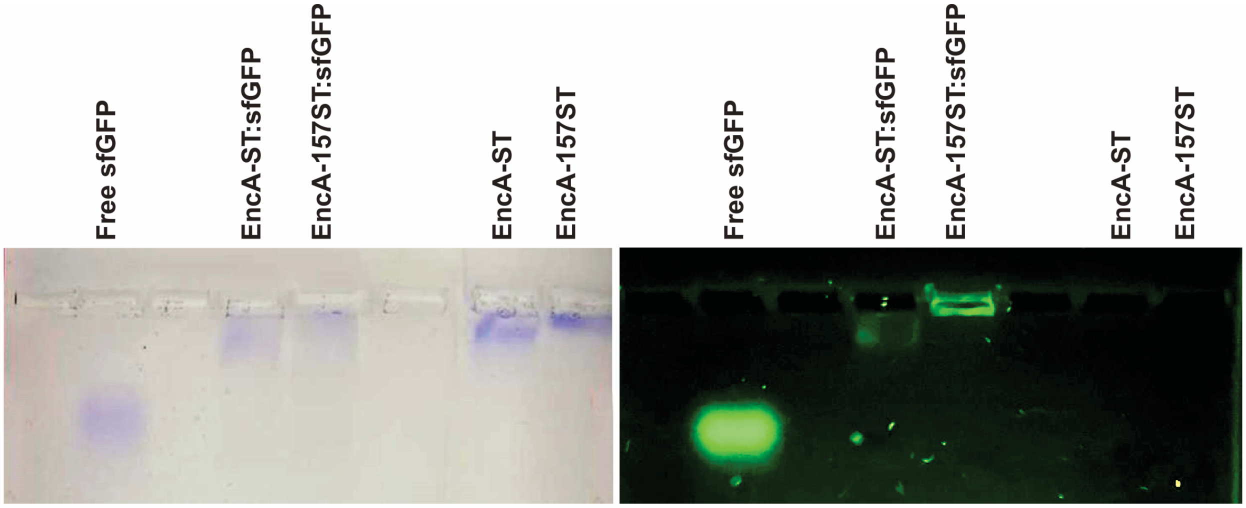

Figure S2. NAGE analysis of the unloaded and sfGFP-CLP loaded encapsulins EncA-ST and EncA-157ST. The free sfGFP-CLP is included as electrophoretic mobility control.

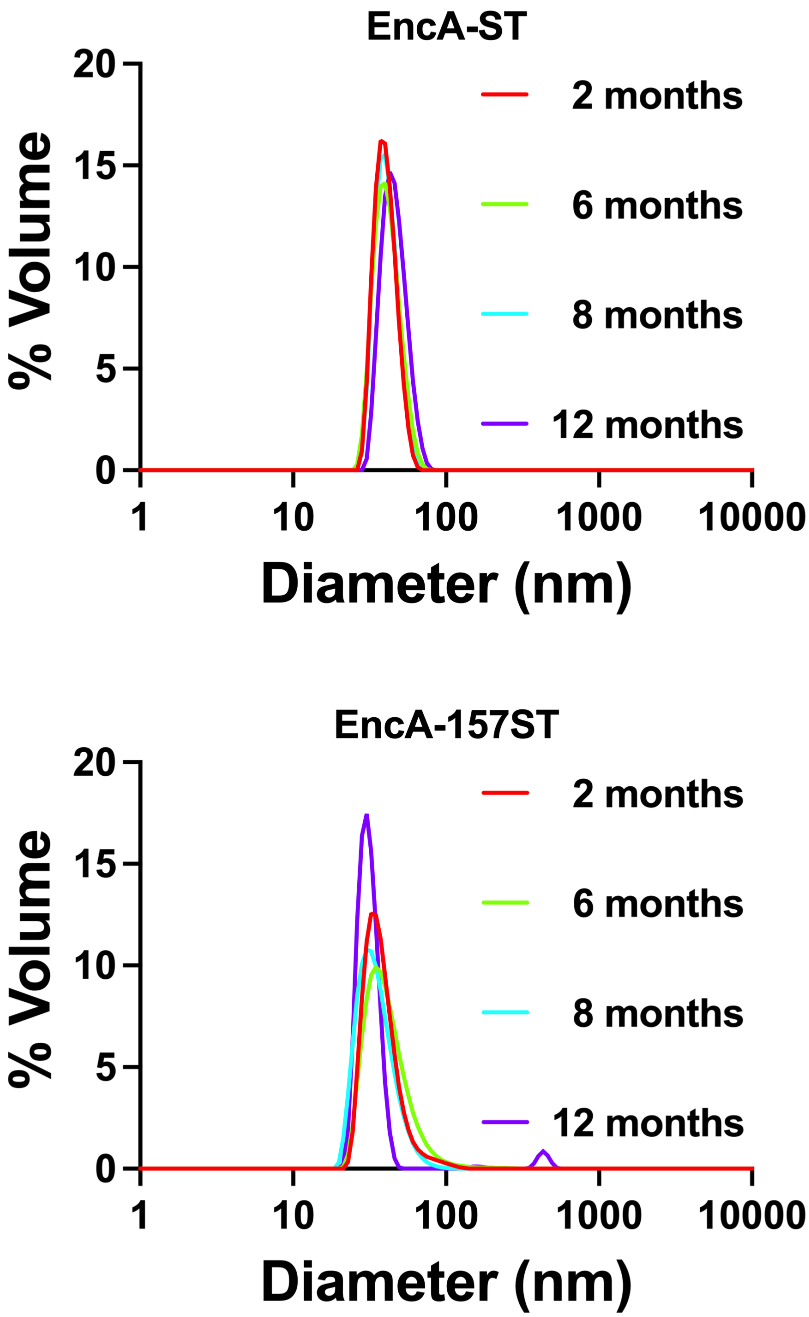

Figure S3. Size of the MxENCs after incubation at room temperature. PDI for the EncA-ST nanocages were 0.129, 0.127, 0.143, and 0.159 for times of 2, 6, 8 and 12 months, respectively). PDI for the EncA-157ST nanocages were 0.293, 0.356, 0.362, and 0.374 for times of 2, 6, 8 and 12 months, respectively).

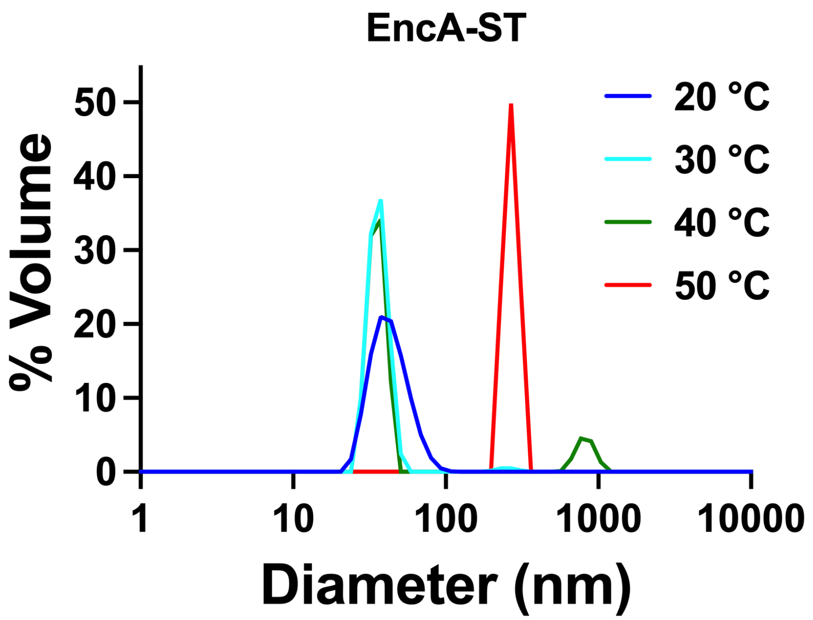

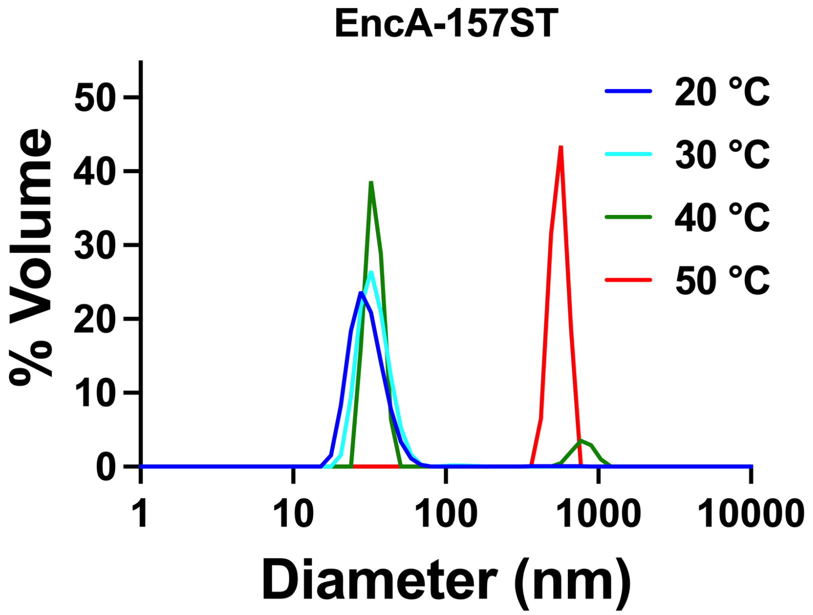

Figure S4. Size of the MxENCs after incubation at different temperatures for 1 h. PDI for the EncA-ST nanocages were 0.156, 0.266, 0.320, and 0.556 for temperatures of 20, 30, 40 and 50 ºC, respectively). PDI for the EncA-157ST nanocages were 0.172, 0.345, 0.415, and 0.581 for temperatures of 20, 30, 40 and 50 ºC, respectively).

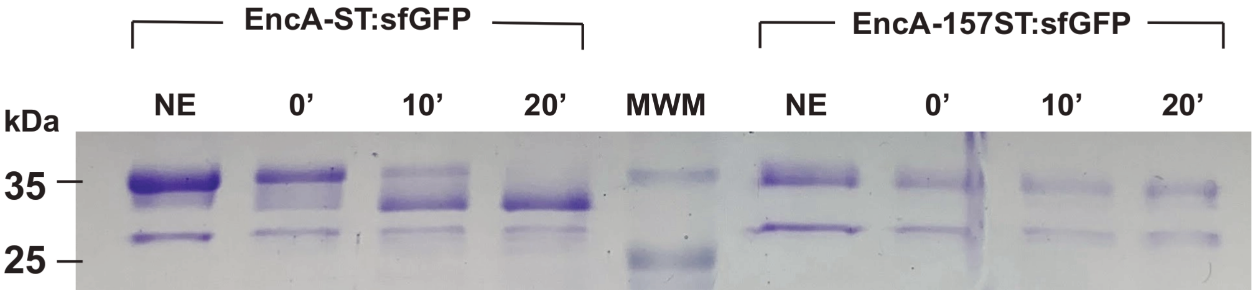

Figure S5. SDS-PAGE analysis of the time course of the proteolytic degradation of the EncA-ST:sfGFP and EncA-157ST:sfGFP complexes incubated with Trypsin for 0, 10 and 20 minutes at 20 ºC and pH 7.4. NE: no enzyme.

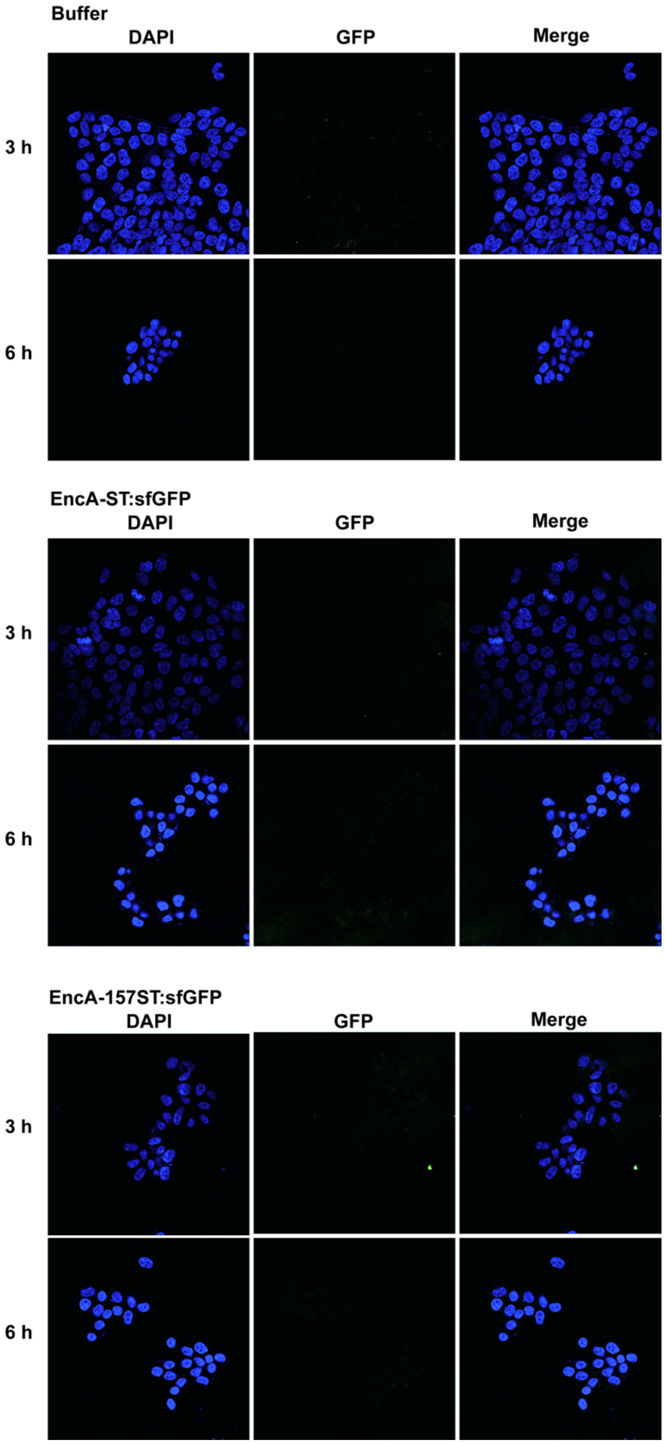

Figure S6. Confocal imaging of HepG2 cells after 3 and 6 hours of treatment. In the first panel, HepG2 cells in DMEM medium were treated with buffer. In the second panel, cells in DMEM medium were treated with 2.5 x10^9 particles/mL of EncA-ST:sfGFP (particles without PreS1 peptide). In the third panel, cells in DMEM medium were treated with 2.5 x10^9 particles/mL of EncA-157ST:sfGFP (particles without PreS1 peptide). DAPI fluorescence (excitation, 504 nm; emission, >523 nm) and GFP fluorescence (excitation, 405 nm; emission, >488 nm).

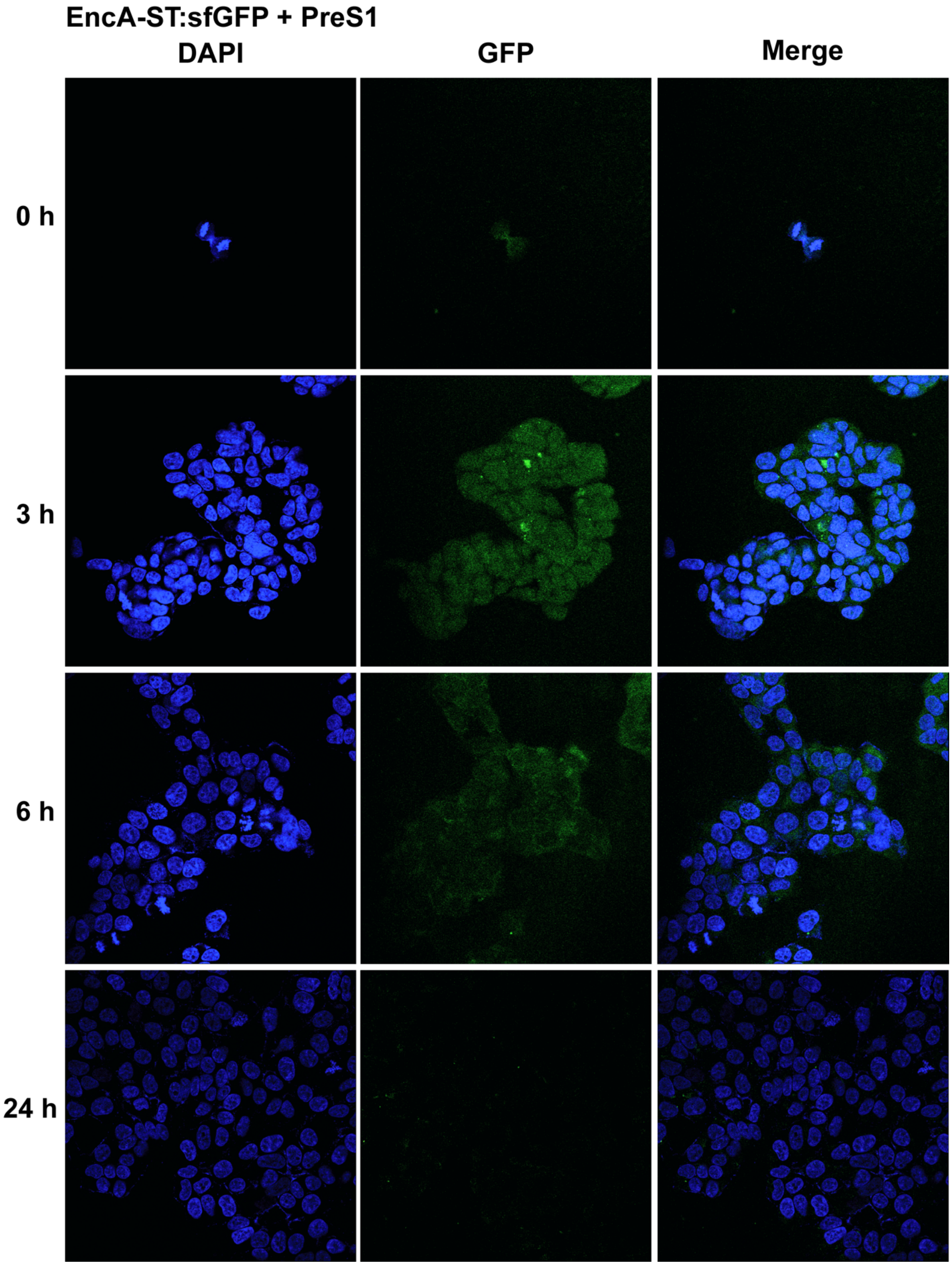

Figure S7. Confocal imaging of HepG2 cells after 0, 3, 6, and 24 hours of treatment with 2.5 x10^9 particles/mL of EncA-ST:sfGFP + PreS1. DAPI fluorescence (excitation, 504 nm; emission, >523 nm) and GFP fluorescence (excitation, 405 nm; emission, >488 nm).

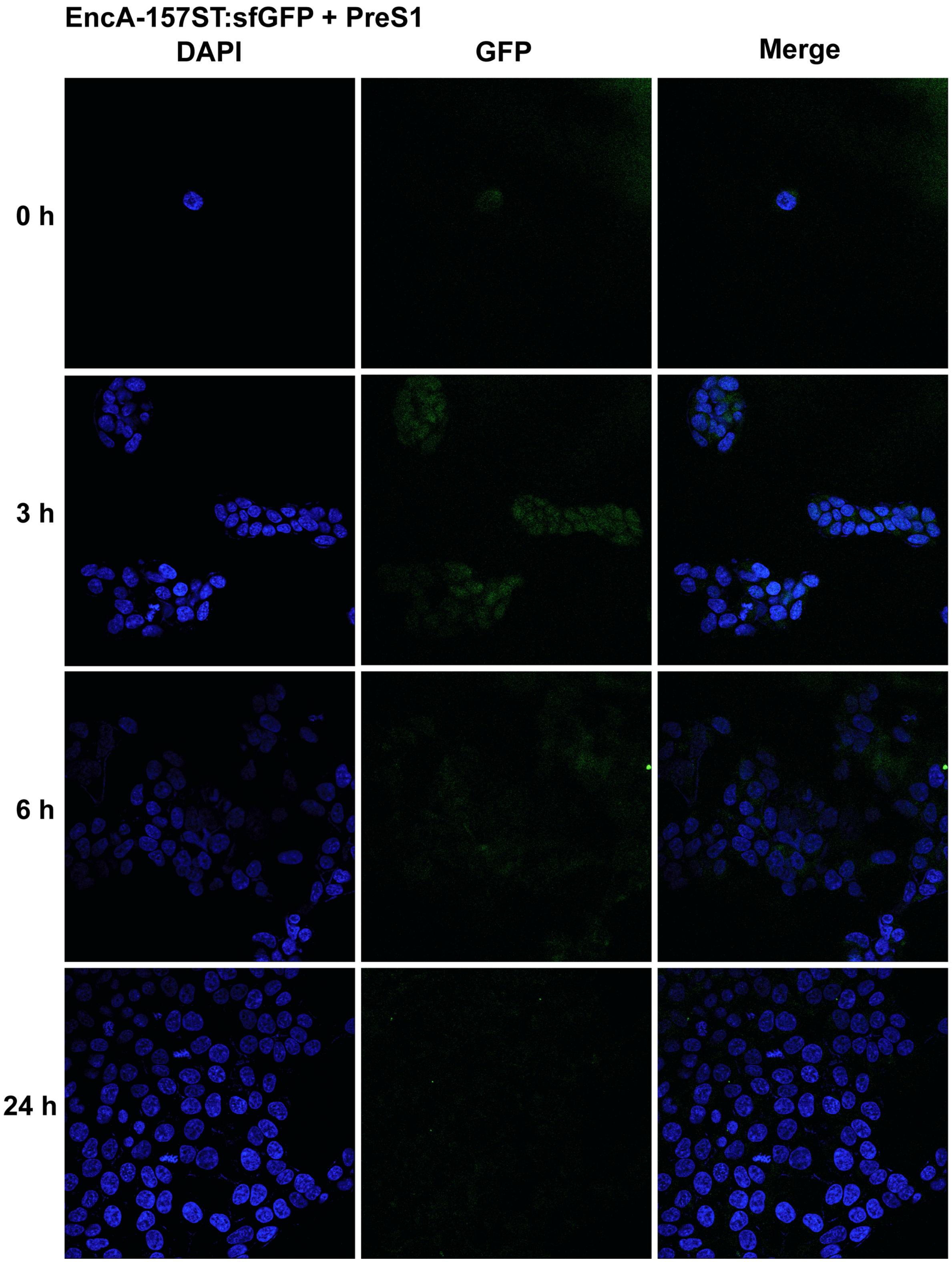

Figure S8. Confocal imaging of HepG2 cells after 0, 3, 6, and 24 hours of treatment with 2.5 x10^9 particles/mL of EncA-157ST:sfGFP + PreS1. DAPI fluorescence (excitation, 504 nm; emission, >523 nm) and GFP fluorescence (excitation, 405 nm; emission, >488 nm).
